# Supplementary material for: Comprehensive Systematic Review of Biomarkers in Metastatic Renal Cell Carcinoma: Predictors, Prognostics, and Therapeutic Monitoring
Source: Cancers (Basel). 2023 Oct 11;15(20):4934. doi: 10.3390/cancers15204934 (PMC10605584; doi:10.3390/cancers15204934)

Supplementary Figure S1: PRISMA flow diagram for new systematic reviews which included searches of databases and registers only

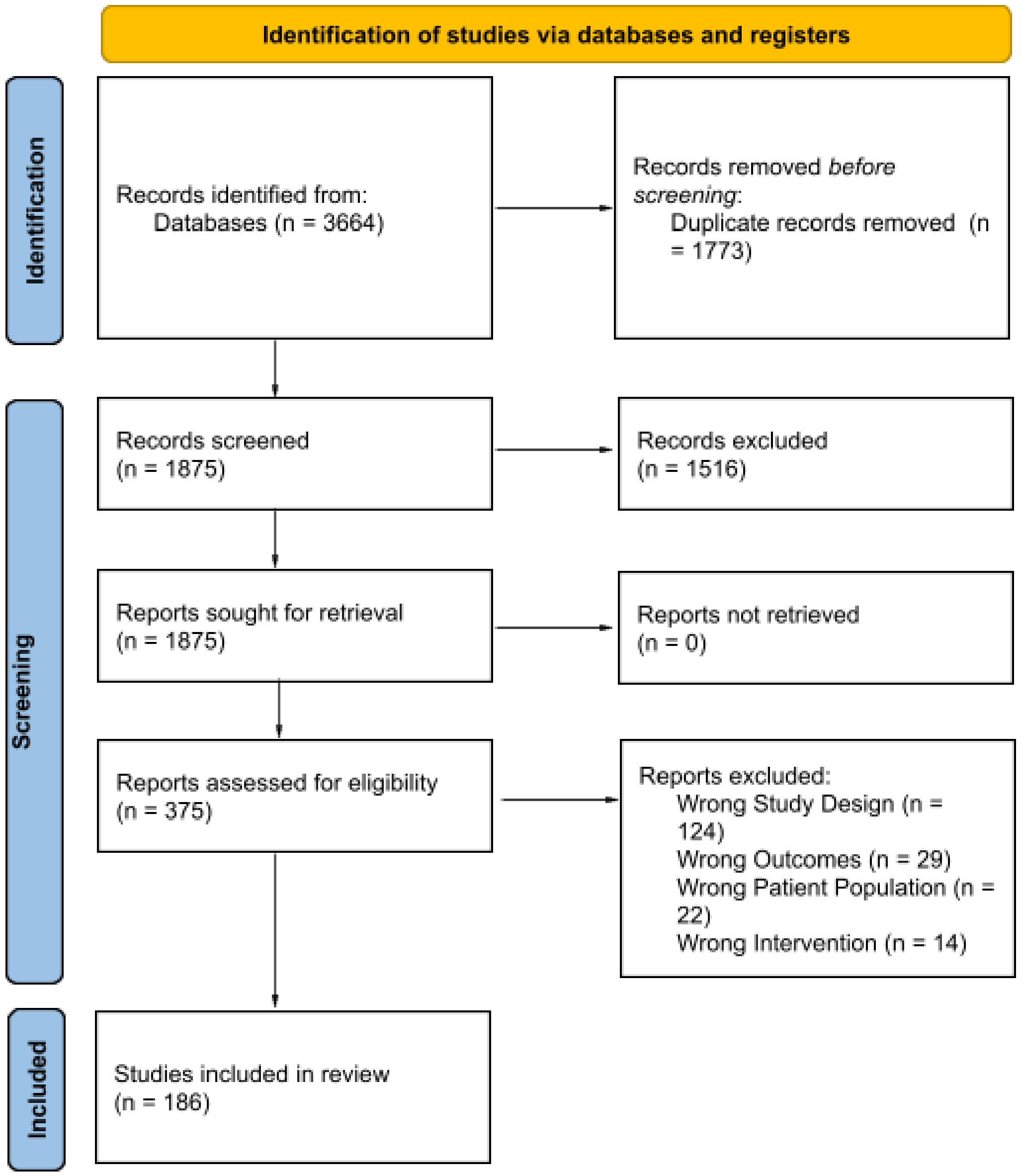

Supplement: Supplementary file 1 [file cancers-15-04934-s001.zip › supplementary Figure 1_ PRISMA Flow Diagram.pdf]
